# Angiosarcoma of gallbladder, a literature review

**DOI:** 10.1186/s13256-023-04323-z

**Published:** 2024-01-31

**Authors:** Mahsa Salehi, Shafi Rehman, Sissmol Davis, Hamid Reza Jafari

**Affiliations:** 1 Mazandaran University of Medical Sciences, Mazandaran, Iran; 2 Khyber Medical College, Peshawar, Pakistan; 3 J.J.M Medical College, Davanagere, India; 4 Isfahan University of Medical Sciences, Isfahan, Iran

**Keywords:** Angiosarcoma, Gallbladder, Epithelioid angiosarcoma, Gastrointestinal tract, Gallbladder angiosarcoma

## Abstract

**Background:**

Angiosarcoma of the gallbladder is a rare diagnostic entity rarely encountered by pathologists and has rarely been reported in literature. This review aimed to examine the clinicopathological features, immunohistochemistry, treatment, and outcomes of gallbladder angiosarcoma.

**Methods:**

A search of the PubMed, Science Direct and Google Scholar was done with the search terms ("angiosarcoma" OR "angiosarcomas") AND ("gallbladder" OR "gallbladders"). Based on inclusion and exclusion criteria, only case reports could be used for this review.

**Result:**

8 case reports were chosen in the end for analysis. The mean age of the patients at presentation was 65 years. It was most frequently observed in males. Abdominal pain and palpable mass were the most commonly reported symptoms. Cholelithiasis and anemia were also reported. On histopathology morphologically epithelioid appearance of angiosarcoma was evident. Cytokeratin (CK) AE1/AE3, Von willebrand factor, Factor VIII antigen, Vimentin, CD31 were positive. Meanwhile, UEA, CD34, CD117, S-100, Keratin, EMA, and CEA showed negative outcome. Surgery was the preferred method of treatment and a mean 10-months follow-up was done.

**Conclusion:**

Despite the unavailability of convincing data, histological and immunohistochemical analyses play a major role in the diagnosis of gallbladder angiosarcoma. Nevertheless, more comprehensive clinical studies are required to provide universal guidelines for the treatment and diagnosis of angiosarcoma of the gallbladder.

## Introduction

Angiosarcoma is a malignancy, vascular tumors, resembling endothelial differentiation in terms of morphology or immunophenotype. It has invasive nature with high likely hood of distal metastasis and poor survival. (1) Roughly, 2–3% of all soft tissue sarcomas in adults are angiosarcomas. (2) Majority of times, etiology is unknown, however, radiation, chronic lymphedema (Stewart-Treves syndrome), exposure to vinyl chloride, arsenic and thorium dioxide (Thorotrast), and surgically implanted foreign materials are some of the risk factors. Moreover, some angiosarcomas are associated with some syndromes such as neurofibromatosis, Maffucci syndrome, and nerve sheath tumors) [1, 3–5].

Majority of angiosarcoma cases stem from head and neck and breast region [6]. On the other hand, angiosarcoma of the gastrointestinal tissue is rare [7, 8]. Furthermore, angiosarcoma of the gallbladder is so rare which all related data is mentioned in the case reports to the best of our knowledge [8–11]. Therefore, in the following article, we have reviewed comprehensively gallbladder angiosarcoma reported cases in regards to demographics characteristics, clinical features, gross findings, histopathology, immunohistochemistry, treatment, and follow-up.

## Materials and methods

### Search strategy

A literature review was performed according to the preferred reporting items for systematic reviews and meta‐analyses statement. A search of the PubMed, Science Direct and Google Scholar was done with the search terms ("angiosarcoma" OR "angiosarcomas") AND ("gallbladder" OR "gallbladders"). The search was completed on October 2023. The results were limited to human‐subject and English‐language articles. All abstracts were analyzed, and full‐ text articles which were open access were obtained when inclusion criteria were fulfilled. Studies and publications with insufficient data or incomplete information were excluded. Additionally, manual search was also performed from the subsequent full‐text articles reviewed to identify additional relevant articles.

### Selection criteria

Our initial intention was to find studies with large sample size. Nonetheless, upon a comprehensive search, we decided to include all case reports due to scarcity of larger studies of angiosarcoma of gallbladder with high sample size. Exclusion criteria were non‐English language, animal, nonobtainable full‐text studies, and studies with insufficient data. A quality assessment tool, the Joanna Briggs Institute (JBI) Critical Appraisal Checklist for Case Reports, was used. The quantitative analysis was performed by combining data in their original metric.

### Data extraction

Variables included author, year of publication, patient demographics, clinical findings, medical history, lab data, radiological and pathological findings, ultra-structural findings, immunohistochemistry, metastasis, treatment, follow-ups and survival data. Data analyses were performed with Microsoft Excel 2018 (Microsoft Corp., Redmond, WA, USA).

## Results

**Fig. 1:**
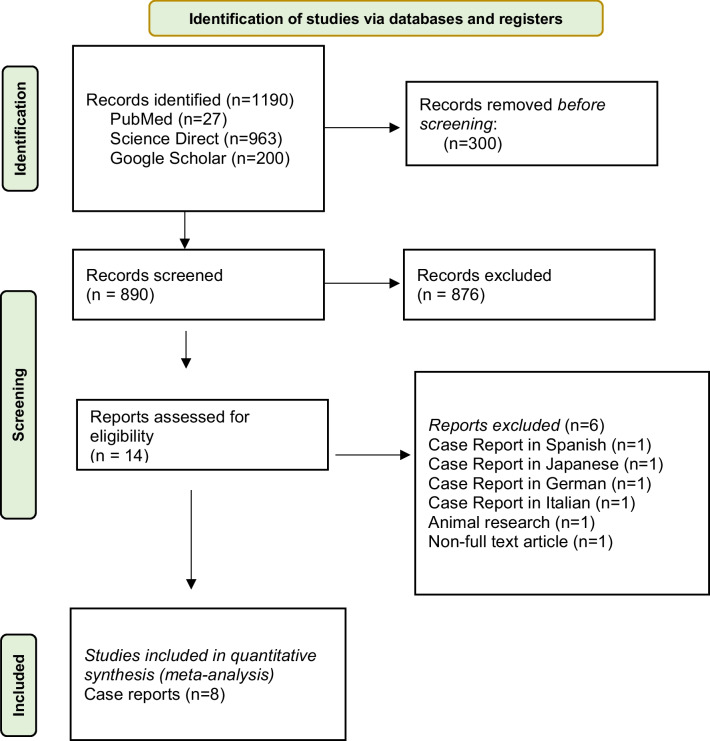
PRISMA flow chart of the extracted articles regarding gallbladder angiosarcoma

Primary search of PubMed, ScienceDirect and Google Scholar search was performed with 890 articles. (Fig. 1) In the end, 8 case reports were included for quantitative synthesis. (Table 1) The results are as follows:

1. Sex

**Table 1:** Summarized data of reported gallbladder angiosarcomas

Reported Cases	Age	Sex	Clinical presentation	Radiological findings	Pathology	Metastasis	Treatment	Follow-up
Rosansky and Mullens [8]	59	M	Abdominal pain and palpable abdominal mass, hepatorenal syndrome without cirrhosis	Gallbladder mass and cholelithiasis in US	Angiosarcoma	yes	Refused Treatment	Died after 28 days of hospitalization (hepatorenal syndrome, renal failure)
Kawai* et al*. [20]	73	M	Abdominal pain and palpable abdominal mass	Gallbladder mass and cholelithiasis in US	Angiosarcoma	yes	Cholecystectomy	Died 4 months after cholecystectomy (cause of death not stated)
Kumar* et al*. [14]	56	M	Palpable abdominal mass	Gallbladder mass and cholelithiasis in US	Epithelioid angiosarcoma	Infiltration of gallbladder mass into the pylorus and omentum	Cholecystectomy with distal partial gastrectomy and gastrojejunostomy	Died 5 months after cholecystectomy (multiple metastases)
Kumar* et al*. [14]	54	M	Palpable abdominal mass	Gallbladder mass and cholelithiasis in US	A squamous cell carcinomaand angiosarcoma	No	Cholecystectomy with wedge resection of the liver combined with a regional lymphadenectomy	Alive 5 years after cholecystectomy
White and Chan [11]	81	F	Abdominal pain and palpable abdominal mass, Fever, Anemia	Gallbladder mass and cholelithiasis in US	Epithelioid angiosarcomas	Peritoneal lesions	Cholecystectomy	Died 2 weeks after cholecystectomy (sepsis)
Costantini* et al*. [9]	57	M	Weakness, Melena, Rectal Bleeding, weight loss, Anemia	Gross echogenic formations and areas of hyper echogenicity caused by lithiasis and thin gallbladder walls in US, CT revealed gross dilation of the gallbladder	A necrotic hemorrhagic appearance. Marked transmural infiltration in gallbladder, epithelioid angiosarcoma, positive factor VIII-related antigen, CD31, and vimentin, negative for epithelial membrane antigen, keratin, CD34, CD117 and S 100	No	Open Cholecystectomy, Secondary laparotomy in 4 months for resection of hepatic segment IV	Alive with no recurrence in 5 months follow-up
Odashiro* et al*. [10]	62	F	Abdominal Pain and nausea and vomiting	Primary acute cholecystitis, Post-op CT of free intraperitoneal blood and amass at the former gallbladder fossa	Epithelial angiosarcoma, von Willebrand factor positive, cytokeratin AE1/AE3 negative	Liver, spleen, ovaries and peritoneal lesions	Open cholecystectomy, secondary laparotomy in 2 months due to hemoperitoneum	Died in 2 months due to hemoperitoneum and hemorrhagic shock
Park* et al*. [15]	78	F	Abdominal pain and Dizziness, Anemia	Primary gallbladder polyp in US, 3.5 cm polyp and focal low-density nodular lesions with pericholecystic infiltration in primary CT scan, an extremely distended and edematous gallbladder with a large hematoma in the lumen in the last CT scan	An intracystic papillary neoplasm (ICPN) with focal adenocarcinoma and angiosarcoma, CD31 positive	No	Ultrasound-guided percutaneous gallbladder drainage followed by laparoscopic cholecystectomy	Alive with no recurrence in 3 months follow-up

Data was available for 8 patients, of whom 37.5% (*n* = 3) were females and 62.5% (*n *= 5) were males.

2. Age

The average was 65 years, with a range from 54 to 81 years. The mean age for females was 73.6 years and for males 59.8 years.

3. Clinical features

Epigastric pain and abdominal mass were the most common symptoms. Moreover, nausea, vomiting, dizziness, melena and rectal bleeding were reported. The mean duration of symptoms were 6.3 weeks with a range of 2 to 12 weeks in general. Cholelithiasis was the most common finding in the past medical history. Anemia was reported in 3 cases.

4. Radiological findings

Computed tomography (CT scan) of abdomen showed distended and edematous gallbladder with a large hematoma in the lumen (*n* = 1), gross dilation of the gallbladder (*n* = 1), and free intraperitoneal blood and a mass at gallbladder fossa (*n*= 1). In addition, ultrasound revealed gross echogenic formations and areas of hyperechogenic (*n* = 1), sonolucent area at the inferior aspect of the right lobe of the liver (*n* = 1), and an echo productive area adjacent to the posterior wall of the gallbladder (*n* = 1).

5. Pathological findings

Macroscopically, gallbladders of two patients showed polypoid masses with gangrenous walls and perforated thick-walled gallbladder surrounded by omental adhesions and abscesses. Transmural infiltration by tumors cells in 2 cases, morphologically epithelioid appearance of angiosarcoma in 3 cases, and mitotic figures in 2 patients were detected. In one of the patients, the neoplastic cells tended to form vascular lacunae containing erythrocytes, with interposition of amyloid-like stroma. Also, mucosa of gallbladder showed extensive ulceration and necrosis. Furthermore, large round to oval vesicular nucleus, a single prominent basophilic nucleolus, intracytoplasmic vacuoles containing erythrocytes, and abundant pale eosinophilic cytoplasm was evident. Regarding findings of another patient, nuclear pleomorphism and hyperchromatism was prominent, particularly in tumor cells lining cleft-like or anastomosing vascular channels angiosarcoma.

6. Immunohistochemistry

Among immunohistochemical stains, Cytokeratin (CK) AE1/AE3 was positive in one case. von Willebrand factor antibodies and Factor VIII antigen were reported positive in one case. CD 31 resembled positive findings in 2 cases. Vimentin was positive in 2 cases. Moreover, UEA, CD34, CD117, S-100, Keratin, EMA, and CEA demonstrated negative result in one of the patients.

7. Metastasis

Out of the data of 8 patients, metastasis was reported in 5 cases. Metastatic sites were peritoneum, abdominal lymph nodes, pancreas, spleen, stomach, intestines, lungs, adrenals, bone, liver, and ovaries.

8. Treatment

Surgical intervention, cholecystectomy, was performed for 7 patients. One patient refused treatment. In one of the cases, pre-operational ultrasound-guided percutaneous gallbladder drainage and resection of hepatic segment IV along with cholecystectomy were performed.

9. Follow-up

The mean follow‐up for patients was found in 8 cases with a mean of 10 months. At the time of the follow-up, 3 patients were alive and 5 were dead. However, prognosis could not be defined.

## Discussion

### Definition

Angiosarcoma is defined as the rapid and extensive infiltrative overgrowth of vascular endothelial cells. It resembles local invasion with high possibility of involvement of lymph nodes and metastasis. High expression of vascular specific receptor tyrosine kinases including TIE1, KDR, TEK, FLT, and VEGF leads to endothelial cell expansion, angiogenesis, and vascular leaks [2, 6, 10, 12, 13].

### Demographic and clinical features

Angiosarcomas of the gastrointestinal tract are such rare neoplasms that their exact incidence is still not known [11, 14]. Angiosarcomas of the gallbladder, according to our knowledge, have been described in 8 case reports [6, 9, 15, 16]. Men were more involved in such cases and the mean age of patients was 65. Association between histological grade and clinical outcomes has been investigated and angiosarcoma is not routinely graded [13, 16–18]. Possibly, cholelithiasis has been advocated for the etiology of gallbladder tumors in general, because of its frequent coexistence with them. Vaittinen* et al* reported cholelithiasis to be present in 79% of sarcomas of the gallbladder [7–11, 14, 16, 19–21]. Though the underlying casus requires deeper establishment, it is said that the irritation due to stones and accompanying inflammation can trigger the development of gallbladder tumors, and hence, angiosarcomas. [22] Clinical symptoms of angiosarcomas of the gallbladder apparently are the same as gallbladder carcinomas. Nevertheless, the diagnosis took shorter period of time as a result of speedy tumoral progression [21].

Diagnosis of angiosarcoma relies on histopathologic examination, since CT scans, ultrasound, or X-rays, can reveals a general suspicion of a tumoral mass. Because of the very limited experience, no definite guideline for treatment exists. As far, surgical intervention remains as the first conventional and reliable treatment. To elaborate more, cholecystectomy with or without wedge resection or extended right hepatic resection, and regional lymph node dissection are performed in majority of cases [22]. Moreover, there is dilemma in including chemotherapy and/or radiotherapy adjacent to surgery in the treatment approach [21]. In one study, age more than 50, tumor larger than 5 cm, and mode of treatment (multimodal versus other) were independent negative prognostic variables [5, 12, 13, 15, 16, 23].

### Pathology

Since a well-differentiated tumor may mimic a hemangioma or hematoma on histology, diagnosis of angiosarcoma cause challenges for clinicians. Detection of endothelial cells with nuclear atypia, abundant mitotic figures, and necrosis could indicate presence of a malignant vascular tumor. The sole histologic variable appearing to have a prognostic implication was mitotic activity. In fact, cases with greater than ten mitoses in high power fields were uniformly fatal within a mean of two years of diagnosis.

On performing immunohistochemistry for ruling out differential diagnosis, vascular markers CD31, CD34 and Factor VIII are specific in differentiating between carcinomas and vascular neoplasms. However, there may be additional difficulties encountered in attempting to differentiate between other neoplasms that stain with vascular markers. Positive immunohistochemical staining for vascular markers may be helpful in differentiating an epithelioid vascular neoplasm from an epithelial neoplasm. Epithelioid angiosarcoma tends to grow in diffuse sheets with larger more pleomorphic cells which contains prominent nucleoli and subtle cleft indicative of vascular differentiation. It is necessary to distinguish Epithelioid hemangioendothelioma (EHE), a vascular neoplasm resembling aggressive behavior and multifocal involvement, from epithelioid angiosarcoma [24]. Moreover, the differential diagnosis of a deep-seated epithelioid neoplasm also consists of malignant melanoma (primary or metastatic), proximal-type epithelioid sarcoma, and epithelioid malignant peripheral nerve sheath tumor. Although such tumors lack true vascular differentiation on histology, immunohistochemistry can play a role in differentiating them. S-100 can be used to ruling out melanoma and epithelioid malignant peripheral nerve sheath tumor [7]. Additionally, CD31 and von Willebrand factor antibodies are helpful in cases of epithelioid angiosarcoma, since it can mimic a poorly differentiated carcinoma on histological investigations. In about one-third of the patients, expression of cytokeratin was positive in epithelioid angiosarcoma. Despite the fact that Weibel Palade bodies presence were negative in angiosarcoma of gallbladder, it played a major diagnostic role in diagnosis of epithelioid angiosarcoma [1, 2, 4–6, 12, 13, 16, 17, 22, 25].

### Follow-up

Based on the findings the included case reports, 3 cases survived the surgery and resembled no recurrence in their follow-ups [2, 9, 14]. On the other hand, one of the patients refused the surgical intervention and died of hepatorenal failure [8]. In 3 of the case reports, patients did not survive due to sepsis, metastasis, hemorrhagic shock in months after their surgeries [10, 11, 21]. Moreover, one of the patients underlying cause of death is unknown [20].

## Conclusions

Our study reviewed all cases of gallbladder angiosarcoma in the literature and summarized their main features. Angiosarcoma of the gallbladder is a rare condition. The relationship of cholelithiasis and gallbladder angiosarcoma requires further clinical investigation. However, mitotic activity was associated with poor prognosis. Despite the scarcity of available data, histological and immunohistochemical analyses appear to play a major role in the diagnosis of angiosarcoma of the gallbladder. Nonetheless, further clinical studies are required to organize a comprehensive universal guideline for the treatment and diagnosis of angiosarcoma of the gallbladder.

## Data Availability

Not applicable.
